# Microneedling in Combination with Topical Pimecrolimus 1% versus Topical Pimecrolimus 1% for the Treatment of Refractory Stable Vitiligo: A Randomized Clinical Trial

**DOI:** 10.1155/2021/5652140

**Published:** 2021-11-30

**Authors:** Fariba Iraji, Ali Asilian, Zahra Talebzadeh, Mina Saber, Fatemeh Mokhtari, Amirhossein Siadat, Seyed Mohsen Hosseini

**Affiliations:** Department of Dermatology, School of Medicine Skin Disease and Leishmaniasis Research Center, Isfahan University of Medical Sciences, Isfahan, Iran

## Abstract

**Objective:**

Vitiligo is a common, autoimmune disease that results in the destruction of the melanocytes and manifests as depigmented macules on various areas of the skin. Numerous treatment options have been proposed for vitiligo. The purpose of this study was to compare the efficacy of microneedling plus topical pimecrolimus 1% versus the sole use of topical pimecrolimus 1% for the treatment of vitiligo.

**Methods:**

This clinical trial was conducted on 30 skin lesions on 15 Al-Zahra hospital patients. Each patient had two similar lesions in the limb area, and each lesion was considered a separate treatment group. The left or right side of the patient's lesion was randomly assigned to receive microneedling plus topical pimecrolimus for three months, while the other side received only topical pimecrolimus 1%. As part of the follow-up, digital photography was taken at the baseline and biweekly for three months after treatment and six months' follow-up. The following methods were used to evaluate the results: DLQI questionnaires, patient satisfaction questionnaires, and two independent dermatologists comparing the improvement rate for each group.

**Results:**

Topical pimecrolimus 1% treatment led to unsatisfactory results, whereas the combination of microneedling and topical pimecrolimus1% treatment produced a more favorable overall outcome (*P* < 0.001).

**Conclusion:**

This study established that combination therapy results in more significant patient improvement. Additionally, one patient experienced mild skin irritation as a side effect of topical pimecrolimus.

## 1. Introduction 

Vitiligo is a common skin disease that affects between 1% and 2% of the world population [1] that occurs equally in men and women and is most prevalent between the ages of 10 and 30 [2]. Although various explanations for the mechanism and etiology of vitiligo have been proposed, including genetics, autoimmune mechanisms, psychological factors, biochemical factors, oxidative stress, and viral infections, the exact cause of vitiligo remains unclear [3, 4].

Multiple factors contribute to the mechanism of vitiligo. Stress, physical and mental trauma, and prolonged exposure to UV light are all possible etiologies of vitiligo [5]. HX positivity is reported in 20%–30% of families [6]. Vitiligo is an autoimmune disease frequently associated with other autoimmune conditions, including hypothyroidism, hyperthyroidism, rheumatoid arthritis, and diabetes mellitus [2]. This disorder is frequently distressing in patients and imposes a cosmetic burden on them, impairing their quality of life, self-esteem, marriage, and employment status [7]. Vitiligo is disfiguring in all races, particularly in dark-skinned people [8]. Vitiligo's basic pathogenesis is currently unknown [9].

While there is no cure for vitiligo, several recommendations include topical steroids, topical calcipotriol, surgery, topical and systemic PUVA, and multivitamins [10, 11]. Calcineurin inhibitors are also a treatment option that appears to have fewer side effects than steroids and are better suited to long-term use, particularly in sensitive areas [12]. Calcineurin inhibitors act as topical immunomodulators by blocking T-cell activation [13]. Microneedling was also used in some studies and was found to be effective in the repigmentation of lesions [14], by transdermal drug delivery as it increases the absorption of topical drugs [15]. Microneedling and topical pimecrolimus have distinct mechanisms of action, and previous research has indicated that combining microneedling with topical treatment may increase the treatment's effectiveness [16]. There has been no comparison of the effects of topical pimecrolimus and microneedling. Thus, this study was conducted to determine the effect of topical pimecrolimus and microneedling on refractory stable vitiligo when used alone or in combination.

## 2. Materials and Methods

### 2.1. Study Design

This study was performed as a randomized clinical trial on 32 lesions from 16 patients who were randomly selected from vitiligo patients who referred to dermatology clinics affiliated with Isfahan University of Medical Sciences between Dec. 2019 and Dec. 2020. After informing all patients of the trial's procedures, written informed consent was obtained, and the trial was initiated. Two similar lesions on two similar limb locations were chosen for each patient. The lesions were then randomly assigned the letters A and B. Lesion A was treated with topical pimecrolimus 1% plus microneedling concurrently, whereas lesion B was treated with topical pimecrolimus 1% solely. Each lesion was examined as a distinct entity. Patients were treated for three months; Microneedling was performed every two weeks, and topical cream was also used twice a day. Patients were then followed up for six months. This study was registered on the Iranian clinical trials registry website under the identification number IRCT20190521043664N. Additionally, this study was approved by Isfahan University of Medical Sciences' bioethics committee and assigned the approval code IR.MUI.MED.REC.1398.413.

The inclusion criteria were patients between the ages of 18 and 60, had vitiligo lesions on their limbs, did not respond to light therapy or topical or systemic treatments, and did not have active vitiligo or new lesions within the past year. The exclusion criteria were pregnancy or breastfeeding, vitiligenous lesions in sites other than extremities, poor compliance to the trial and follow-up, disease deterioration during the study, current or previous vitiligo treatment one year prior to enrollment, keloid tendency, systemic disease, and severe allergic reactions or side effects to the drugs used throughout the trial.

**Figure 1:**
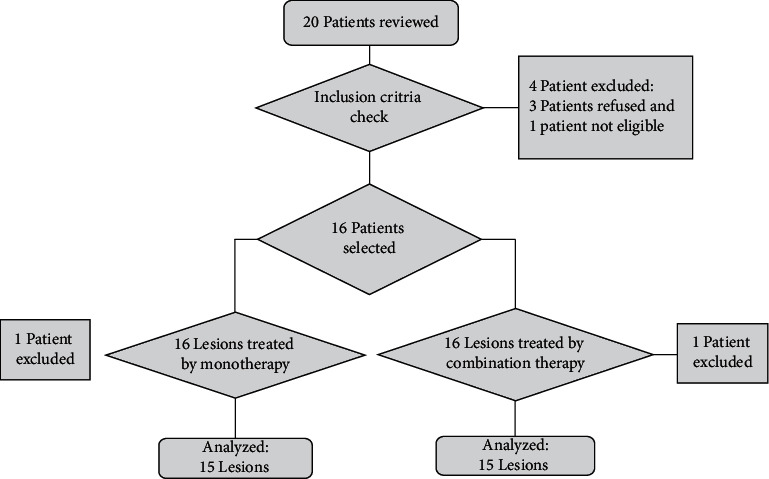
The flowchart of the study.

Initially, the inclusion criteria were used to evaluate and confirm the qualifications of 20 patients. Four patients were excluded from the study during the first stage because three were unwilling to participate and another was ineligible. Thus, 32 lesions were chosen from 16 patients with stable refractory vitiligo. Three months of treatment and six months of follow-up were required. One patient was excluded from the study after the first session due to personal reasons; hence, the final analysis included 30 lesions from 15 patients (Figure 1).

### 2.2. Procedure Steps

Lesions A was simultaneously treated with topical pimecrolimus 1% and microneedling, and topical treatment with pimecrolimus cream 1% was initiated twice daily and continued for three months for lesions classified as B. Microneedling was used to treat these lesions every two weeks, and topical pimecrolimus 1% was applied immediately to the lesion under an occlusive dressing. The amount of topical pimecrolimus ointment was such that it covered the surface of the lesions in a thin layer. After 48 hours, patients were instructed to remove the dressing and apply topical pimecrolimus cream 1% twice daily.

After sterilizing the lesion site, the patient was given local anesthesia for one hour using Xyla-P topical cream (Tehran Shimi, Tehran, Iran) under closed dressing, and subsequently, microneedling was performed. Then, the predetermined lesion site was prepared and draped.

**Figure 2:**
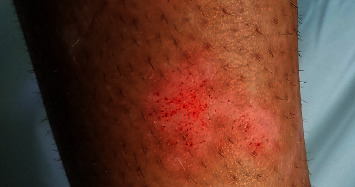
20-year-old female patient's right leg: the appearance of pinpoint bleeding, the endpoint of the microneedling procedure.

Microneedling was conducted with Med Amiea revive (MT.DERM GmbH, Berlin, Germany). The needle penetration depth was initially set to 0.5 mm and then increased gradually until the lesion observed pinpoint bleeding. The microneedling procedure involves a combination of horizontal, vertical, and oblique device passes on the selected lesions, repeating approximately 3 to 4 times, till pinpoint bleeding has appeared (Figure 2). The pen was held vertically against the skin, and the bleeding spot was removed using sterile gauze impregnated with normal sterile saline. Since the microneedling was used for further penetration of topical cream, the treatment was performed just in the lesional area.

Microneedling is considered a noninvasive esthetic procedure with a low rate of associated side effects. But, skin irritation, mild erythema, swelling, dryness, and flaking of the skin could be reported [17]. Also, burning, stinging, itching, and swelling are considered as the most common side effects of pimecrolimus. So, it was instructed to the patients to report in case of any side effects [18].

### 2.3. Treatment Evaluation

The first step was to photograph patients using standardized global photography. All photos were taken with a digital camera (Canon IXUS 185 Digital Camera) in the same room and under the same lighting conditions regarding background, angle, and distance from the light source. Two independent dermatologists who were blinded to the study reviewed the baseline and posttreatment photos. The following qualitative responses to repigmentation were recorded: (0%–25%): mild improvement, (26%–50%): moderate improvement, (51%–75%): good improvement, and (76%–100%): excellent improvement and follicular repigmentation [19].

Patients were evaluated using a patient satisfaction questionnaire, the dermatology quality of life index (DQLI) [8], and repigmentation responses to treatment as determined by two unbiased dermatologists blinded to the study [19].

### 2.4. Statistical Analysis

SPSS statistical software version 24 was used to analyze the data (IBM, Armonk, NY, USA). Mean and standard deviation were used to represent quantitative data, while frequency or percentage represented qualitative data. The chi-square test was used to compare the data. *P* values less than 0.05 were considered statistically significant in all analyses.

## 3. Results

Fifteen objects, eight (53.33%) males and seven (46.66%) females, were followed up in this intervention.  Table 1 summarizes the patients' demographic characteristics.

**Figure 3:**
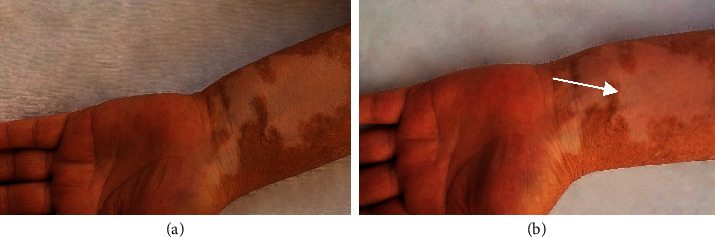
A 47-year-old female patient with refractory patches on the dorsal aspect of her left forearm: (a) before treatment; (b) six months' follow-up after treatment by topical pimecrolimus 1% on the pointed lesion. No changes were observed.

**Figure 4:**
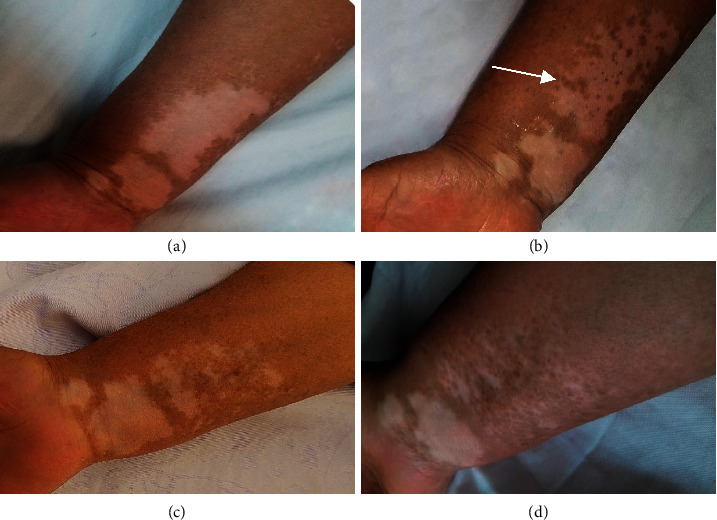
(a) A 47-year-old female patient with three refractory patches on the dorsal aspect of her right forearm, before treatment, (b) two months after treatment by microneedling plus topical pimecrolimus 1% on the pointed lesion (the largest lesion of the three), (c) three months after treatment with same procedure, and (d) at six months' follow-up after treatment.

Table 2 shows that no significant improvements were observed in the pimecrolimus method during the treatment or follow-up period (Figure 3), while in the combination therapy, the amount of repigmentation increased from the start to the end of the treatment and follow-up period. In combination therapy, we had poor recovery levels after two weeks of treatment and fair repigmentation reached to 40% after two months. Also, 33.3% of patients showed a good recovery status three months after treatment and excellent repigmentation appeared in 6.7% after 6 months' follow-up (Figure 4).

Friedman's test demonstrated that patients' recovery status improved significantly over time when microneedling and pimecrolimus were combined (*P* < 0.001). Fisher's exact test revealed that the frequency distribution of patients' recovery levels was not significantly different between the two groups after one month of treatment (*P*=0.999), but there was a noticeable difference at other points of time (*P* < 0.05).

As depicted in Table 3, no patient was satisfied with the pimecrolimus method from the start to the end of the study. In the combination method, there was no significant patients' satisfaction until 45 days after treatment. But, after 3 months' and 6 months' follow-up, 26.7% and 33.3% of respondents, respectively, reported a moderate and high level of satisfaction.

Friedman's test demonstrated that patient satisfaction with the combination of microneedling and pimecrolimus (*P* < 0.001) methods significantly increased over time. Fischer's exact test revealed a statistically significant difference in patient satisfaction at 45 days, two months, 75 days, three months, and six months following treatment in two groups.

Table 4 summarizes the numerical indices of patients' DLQI scores before treatment until the end of 6 months of follow-up. The Kruskalovalis test indicated no significant difference in the DLQI scores of the two groups throughout the study (*P* > 0.05). Friedman's test revealed that when microneedling and pimecrolimus were used in combination, DLQI scores decreased significantly (*P*=0.002).

There were no side effects reported in fourteen patients. Burning was observed in one patient following topical pimecrolimus, but the statistical analysis was not possible due to the complication's low frequency. No side effects were observed following the use of the microneedling device, and no infection, edema, or severe pain was reported.

## 4. Discussion

Vitiligo is a common, autoimmune disease that results in the destruction of the melanocytes and manifests as depigmented macules on various areas of the skin [20]. Despite the abundance of research and therapeutic modalities, there is still no cure for this condition [21, 22]. The patients' therapeutic approach should be personalized and tailored to their age, race, vitiligo subtype, affected skin area, and disease stability [23].

Topical calcineurin inhibitors (TCIs), such as tacrolimus and pimecrolimus, are widely used in vitiligo treatment [5]. On the other hand, the efficacy of microneedling for the treatment of vitiligo has been suggested in previous studies [24]. Microneedling is a minimally invasive procedure that has been used to treat a variety of dermatological conditions. Studies have shown that microneedling is a safe and effective treatment method for vitiligo, and its combination with other methods such as topical tacrolimus, topical calcipotriol, and 5-fluorouracil has demonstrated increased efficacy [24]. In the current study, we evaluated and compared the efficacy of topical pimecrolimus cream 1% alone with a combination of microneedling plus topical pimecrolimus 1% in the treatment of the patients with resistant vitiligo.

The efficacy of pimecrolimus alone cream for the treatment of the head and neck vitiligo has been shown by Boone et al. These researchers evaluated the efficacy and safety of pimecrolimus cream 1% in treating 26 patients with vitiliginous lesions in the head and neck and reported a median repigmentation rate of 72.9% after a six-month treatment period [25]. The present study demonstrated that combining microneedling with topical pimecrolimus effectively treats refractory stable vitiligo and improves patients' satisfaction. This higher response rate in the combination group is possibly due to the penetrance enhancer effect of microneedling for pimecrolimus as a synergetic effect or additive effect of the 2 treatment methods.

Dawid et al. reported pimecrolimus cream 1% ineffectiveness for treating body lesions of vitiligo. The authors reported that no improvement was observed in lesions after six months of treatment by topical pimecrolimus [26]. Consistent with this study, our findings showed none-efficacy of pimecrolimus cream alone for treatment of the limb lesions; however, the use of microneedling in addition to pimecrolimus provided a positive effect.

Mina et al. compared the efficacy of microneedling combined with 5-fluorouracil vs. microneedling combined with tacrolimus. The authors reported starting of the clinical repigmentation after three sessions of microneedling (i.e., within six weeks) [27]. In our study, as aforementioned, all patients in the pimecrolimus and pimecrolimus plus microneedling groups demonstrated a poor response within the first 45 days of the trial and it was after approximately two months that patients in the combination group showed clinical repigmentation.

A systematic review and meta-analysis showed that monotherapy with TCIs has favorable outcomes, with 55% of patients achieving a mild response during a three-month treatment period. Additionally, at least 89.5% showed a mild response to the combination therapy of TCIs and phototherapy [28]. The results of our study also showed the superiority of the combination method over pimecrolimus monotherapy although instead of phototherapy, we used microneedling as the adjoint method.

In this study, the vitiligo lesions did not respond to topical pimecrolimus 1% monotherapy. This could be explained partly by the fact that these lesions were located on the extremities, which are more resistant to therapy than the rest of the body [29]. It should also be mentioned that we only selected lesions that were resistant to previous topical or systemic therapy.

Overall, our results showed the higher response rate of topical pimecrolimus plus microneedling combination versus pimecrolimus cream alone method as in terms of skin repigmentation. In addition, no significant side effect was observed in the patients of both groups that could be considered as a promising finding. To our best knowledge, this is the first study that evaluated a combination of microneedling plus pimecrolimus in the treatment of vitiligo. The present study demonstrated that combining microneedling with topical pimecrolimus effectively treats refractory stable vitiligo and improves patient satisfaction.

### 4.1. Study Limitation

Because of the COVID-19 pandemic, we had a small sample size.

## 5. Conclusions

Our study showed the efficacy and safety of microneedling plus pimecrolimus cream for the treatment of the resistant vitiligo lesions of the limbs. Further studies with a larger sample size are recommended to better evaluate the efficacy of this method.

## Figures and Tables

**Table 1:** Clinical data of studied cases.

		No.	%
Sex	Male	8	53.33
	Female	7	46.66

Age (years)	Mean ± SD	33.8 ± 10.10	
	Min-max	18–56	

Family history	Negative	12	80.00
	Positive	3	20.00

Type of skin	III	10	66.66
	IV	5	33.33

Previous history of treatment	Yes	15	100
	No	0	0

**Table 2:** Comparison between topical pimecrolimus therapy and combination therapy according to degree of repigmentation during the treatment and follow-up period.

Treatment method	Pimecrolimus	Combination of microneedling and pimecrolimus				Fischer's exact test
Repigmentation status	Poor	Poor	Fair	Good	Excellent	
After 2 weeks	15 (100%)	15 (100%)	0 (0%)	0 (0%)	0 (0%)	—
After 1 month	15 (100%)	14 (93.3%)	1 (6.7%)	0 (0%)	0 (0%)	0.999
After 45 days	15 (100%)	11 (73.3%)	4 (26.7%)	0 (0%)	0 (0%)	0.027
After 2 months	15 (100%)	9 (60%)	6 (40%)	0 (0%)	0 (0%)	0.01
After 75 days	15 (100%)	6 (40%)	7 (46.7%)	2 (13.3%)	0 (0%)	0.001
After 3 months	15 (100%)	6 (40%)	4 (26.7%)	5 (33.3%)	0 (0%)	<0.001
After 6 months' follow-up	15 (100%)	6 (40%)	4 (26.7%)	4 (26.7%)	1 (6.7%)	<0.001
Friedman test	—	<0.001				

**Table 3:** Percentage of patients' satisfaction in both groups during treatment and follow-up.

Treatment method	Pimecrolimus	Combination of microneedling and pimecrolimus				Fischer's exact test
Patients' satisfaction	Ultralow	Ultralow	Low	Moderate	High	
After 2 weeks	15 (100%)	14 (93.3%)	0 (0%)	1 (6.7%)	0 (0%)	0.999
After 1 month	15 (100%)	13 (86.6%)	1 (6.7%)	1 (6.7%)	0 (0%)	0.762
After 45 days	15 (100%)	10 (6.66%)	1 (6.7%)	4 (26.7%)	0 (0%)	0.035
After 2 months	15 (100%)	9 (60%)	1 (6.7%)	5 (33.3%)	0 (0%)	0.019
After 75 days	15 (100%)	6 (40%)	0 (0%)	7 (46.6%)	2 (13.4%)	<0.001
After 3 months	15 (100%)	6 (40%)	0 (0%)	4 (26.7%)	5 (33.3%)	<0.001
After 6 months' follow-up	15 (100%)	6 (40%)	0 (0%)	4 (26.7%)	5 (33.3%)	<0.001
Friedman test	-	<0.001				

**Table 4:** Comparison of numerical indices of patients' DLQI scores in monotherapy and combination therapy.

Treatment method	Pimecrolimus			Combination of microneedling and pimecrolimus			Kruskalovalis test
DLQI	Mean standard deviation	Median	Interquartile range	Mean standard deviation	Median	Interquartile range	
Before treatment	20.50 (1.65)	20.50	(22, 19)	20.73 (1.83)	21	(22, 19)	0.937
After 2 weeks	20.64 (1.59)	21	(22, 19)	20.73 (1.62)	21	(22, 19)	0.953
After 1 month	20.67 (1.23)	21	(22, 20)	20.33 (1.49)	21	(22, 19)	0.812
After 45 days	20.80 (1.32)	21	(21, 20)	20.20 (2.04)	20	(22, 19)	0.699
After 2 months	20.67 (1.49)	21	(22, 19)	20.00 (2.03)	20	(21, 18)	0.492
After 75 days	21.00 (1.51)	21	(22, 20)	19.07 (2.57)	19	(21, 18)	0.091
After 3 months	20.80 (1.47)	20	(22, 20)	18.47 (3.42)	18	(22, 15)	0.132
After 6 months' follow-up	20.80 (1.74)	21	(22, 19)	18.47 (3.46)	18	(22, 15)	0.149
Friedman test	0.943			0.002			

## Data Availability

The data used to support the findings of this study are included within the manuscript.
